# *TEMPy*2: a Python library with improved 3D electron microscopy density-fitting and validation workflows

**DOI:** 10.1107/S2059798320014928

**Published:** 2021-01-01

**Authors:** Tristan Cragnolini, Harpal Sahota, Agnel Praveen Joseph, Aaron Sweeney, Sony Malhotra, Daven Vasishtan, Maya Topf

**Affiliations:** a Institute of Structural and Molecular Biology, Birkbeck, University College London, London, United Kingdom; b Oxford Particle Imaging Centre, Division of Structural Biology, Wellcome Trust Centre for Human Genetics, University of Oxford, Oxford, United Kingdom; c Centre for Structural Systems Biology, Heinrich-Pette-Institut, Leibniz-Institut für Experimentelle Virologie, Hamburg, Germany; d Universitätsklinikum Hamburg Eppendorf, Hamburg, Germany

**Keywords:** three-dimensional electron microscopy, model fitting, validation, fitting scores, model assessment, macromolecular complexes, *TEMPy*2

## Abstract

Structural determination of molecular complexes by cryo-EM requires large, often complex processing of the image data that are initially obtained. Here, *TEMPy*2, an update of the *TEMPy* package to process, optimize and assess cryo-EM maps and the structures fitted to them, is described. New optimization routines, comprehensive automated checks and workflows to perform these tasks are described.

## Abstract 2

*TEMPy*2, an update of the *TEMPy* package to process, optimize and assess cryo-EM maps and the structures fitted to them, is presented.

## 1. Introduction   

Structural determination of biological assemblies is paramount to understanding their function. Cryo-EM is experiencing an exponential growth in popularity, in particular owing to its ability to resolve large assemblies in an aqueous environment without the need for crystallization. This has allowed large structures to be resolved quickly, with the recent coronavirus spike-protein structure determinations being a salient example (Wrapp *et al.*, 2020 ▸).

Pharmaceutical applications are also becoming more common, as cryo-EM can be used to not only determine alternate conformations (*e.g.* with and without ligand), but also to determine the position and mechanism of ligand binding (Atherton *et al.*, 2017 ▸). Although the applications of cryo-EM are widespread, the data-processing step is paramount to obtain good and reliable structural information (Vinothkumar & Henderson, 2016 ▸; DiMaio *et al.*, 2013 ▸).

Despite the so-called ‘resolution revolution’ (Kühlbrandt, 2014 ▸), many of the recently solved structures of biological assemblies still suffer from having a resolution that is far from near-atomic, especially in peripheral and flexible regions of the structure, where it may be difficult to unambiguously place side chains, backbone or even entire subunits. This is particularly salient for large biological complexes, where the assembly may be dynamic, rendering the placement of sub­units ambiguous (Kim *et al.*, 2018 ▸; Zhou *et al.*, 2019 ▸).

The refinement of an initial model may also leave regions that poorly match the density, which may not be readily captured by global scoring methods. Overfitting is also a common, but hard to detect, issue during model reconstruction (Chen *et al.*, 2013 ▸).

As cryo-EM becomes ever more popular, a modern toolkit that allows users to compare and optimize maps and the structures fitted to them is of great importance (de la Rosa-Trevín *et al.*, 2016 ▸; Burnley *et al.*, 2017 ▸). We present an update of *TEMPy*, a Python-based package that allows users to process cryo-EM maps and the structures associated with them (Farabella *et al.*, 2015 ▸). We have developed a new version of the package, and present herein the improvements built into it. As ever more complex tasks are automated in packages such as *TEMPy*2, quality control upon code changes becomes critical to ensure the reproducibility and correctness of the results (Wilson *et al.*, 2017 ▸). We present the improvements that are now built into the *TEMPy*2 codebase to reach this goal. After going over the package organization and its content, we will show examples of workflows for common tasks performed on EM data sets using the package.

## 2. Package organization   

The* TEMPy*2 code can be found at http://tempy.ismb.lon.ac.uk and in the PyPI package repository at https://test.pypi.org/project/BioTEMPy.

The code is divided into subpackages, each targeted towards common tasks performed on EM maps and related structures:

(i) the *maps* subpackage handles the creation and manipulation of maps;
(ii) the *protein* subpackage handles the manipulation of structural data and its comparison to maps;
(iii) the *math* subpackage handles operations such as geometric transforms used on atomic structures as well as maps;
(iv) the *assembly* subpackage deals with the optimization of multiple structures within a map;
(v) the *graphics* module contains routines to generate plots from the data produced by various analyses.

**Figure 1:**
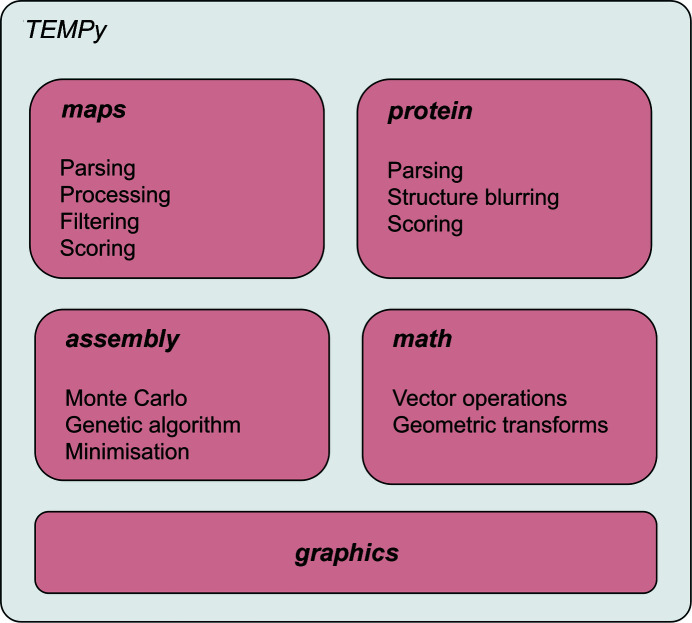
Depiction of the package organization.

Fig. 1 ▸ provides a visual representation of the package organization.

Several tools built on *TEMPy*2 routines with command-line interfaces are present that make use of these routines. These include *γ-TEMPy*, ‘local fit optimizer’ and ‘local fit quality estimation’, among others.

### 2.1. Scoring functions   

#### 2.1.1. Global scoring   

Several scoring functions are implemented, as well as routines to transform the data as required. Cross-correlation coefficient calculations, for example, can be performed either between two maps or between a map and a structure blurred to a given resolution level.

**Figure 2:**
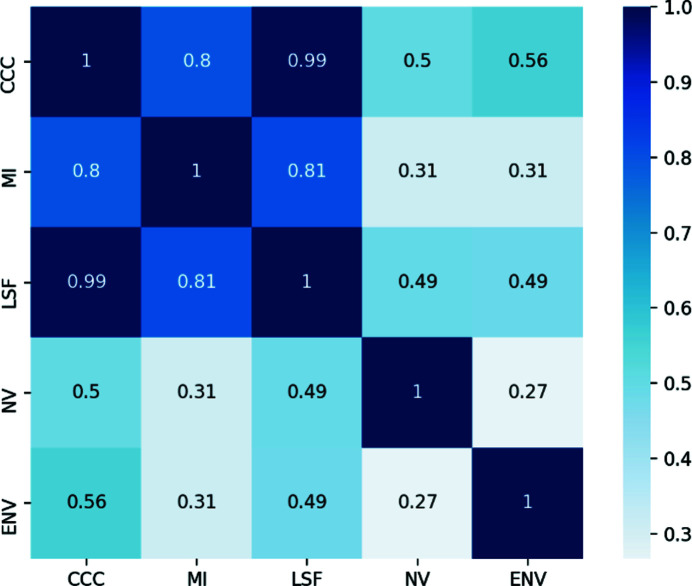
Correlation of scores available within *TEMPy*2. The low correlation between some of the scores indicates that they rank the quality of fit between two maps or a map and structure in qualitatively different ways.

There are different global scores available in *TEMPy*2 (Table 1 ▸) that include cross-correlation coefficient (CCC; Roseman, 2000 ▸), mutual information (MI; Vasishtan & Topf, 2011 ▸), least-squares fit (LSF; Vasishtan & Topf, 2011 ▸), normal vector scores (NV; Vasishtan & Topf, 2011 ▸) and envelope score (ENV; Vasishtan & Topf, 2011 ▸). Fig. 2 ▸ provides an estimate of the correlation between the scores, which has been computed across a data set of 155 structures for the same CASP target T0984 (qualitatively similar results are obtained on other data sets).

While CCC is the standard measure, MI may be a better measure at lower resolution and when the noise level is higher (Vasishtan & Topf, 2011 ▸; Joseph *et al.*, 2017 ▸).

### 2.2. Local scoring   

Local scores have been developed to provide a measure of the quality of fit for different parts of a model. While model building and refining a fitted model to a map, the quality of the fit is rarely homogeneous: certain regions are better fitted to the map, and the map itself may not resolve all features with the same resolution (Cardone *et al.*, 2013 ▸). Local scores are therefore paramount to discover and understand which regions should be the focus of further refinement. We present below two local scores present in *TEMPy*2.

The segment-based cross-correlation coefficient (SCCC) is a local measure that can be applied at any level, for example domain, subdomain and secondary-structure element. The segment-based Mander’s overlap coefficient (SMOC), on the other hand, is a correlation measure at the residue level only. SCCC may provide better results at lower resolution or for maps that are significantly different, while SMOC may be better for higher resolution maps or to compare similar maps (Joseph *et al.*, 2017 ▸).

A correlation matrix of the different local scores has been obtained for structure and models generated during the CASP13 competition, including SCCC, SMOC, EMRinger and other existing local scores (see Fig. 3 of Kryshtafovych *et al.*, 2019 ▸).

#### 2.2.1. Segment-based cross-correlation coefficient (SCCC)   

The SCCC provides a measure of the quality of fit of different segments (Pandurangan *et al.*, 2014 ▸).

This can be useful to identify segments that require better fitting in the density and could be refined using flexible fitting approaches (Joseph *et al.*, 2016 ▸).

#### 2.2.2. Segment-based Mander’s overlap coefficient (SMOC)   

The SMOC score gives a sequence-based local estimate of the fit quality of an atomic model to a map (Joseph *et al.*, 2016 ▸). The algorithm computes Mander’s overlap coefficient over the local region around each residue.

Two variants are available.

(i) In SMOC_f_ the local region encompasses all voxels covered by residues in a sequence window centred at the residue of interest. The size of the window can be adjusted based on the map resolution.
(ii) In SMOC_d_ the local region covers voxels within a distance from atoms of a residue. The distance is automatically adjusted based on the map resolution. This recently introduced variant is included in the current update.

### 2.3. Unit tests   

For any evolving scientific package that is designed to handle and analyse data, it is important to ensure the correctness and self-consistency of the produced results as code improvements and new functionalities are introduced. We have introduced thorough automated checks in our code base, in the form of unit tests for most routines, as well as more complex, full-fledged practical tests using input data. 16 of the 36 modules have full coverage and 28 have partial coverage, with a per-function coverage of 34%.

### 2.4. Python version change   

As Python version 2 has now been deprecated (https://www.python.org/doc/sunset-python-2/), it is important to ensure that newer code can be developed that still makes use of *TEMPy*2. Therefore, we have moved the code base to Python version 3. While the syntaxes of both are highly similar, subtle changes may cascade, causing errors in the data handling. The unit tests we have incorporated have allowed us to check and adjust the code accordingly, and ensure consistent handling across version changes, for example by making sure that an optimization in the CCC calculation does not change the values returned, or that a change in map loading results in the same voxel values as before.

### 2.5. Input data   

The Protein Data Bank (PDB; Burley *et al.*, 2019 ▸) has issued recommendations to move to the newer, less ambiguous mmCIF format to store and manipulate structural data (Adams *et al.*, 2019 ▸). The current version of *TEMPy*2 now handles both the legacy PDB format as well as the newer CIF data.

Similarly, thorough checking has been conducted to ensure near-complete compliance with the 2014 norm for the MRC format produced by CCP-EM (Cheng *et al.*, 2015 ▸).

#### 2.5.1. Compression   

The map data used in cryo-EM can occupy a large amount of disk space and are often stored and provided in a compressed format. To make it more practical to handle these data, *TEMPy*2 has been rewritten to natively handle gzip compression and decompression, allowing those files to be manipulated without prior manual decompression or requiring recompression after manipulation with *TEMPy*2.

## 3. Workflow examples   

**Figure 3:**
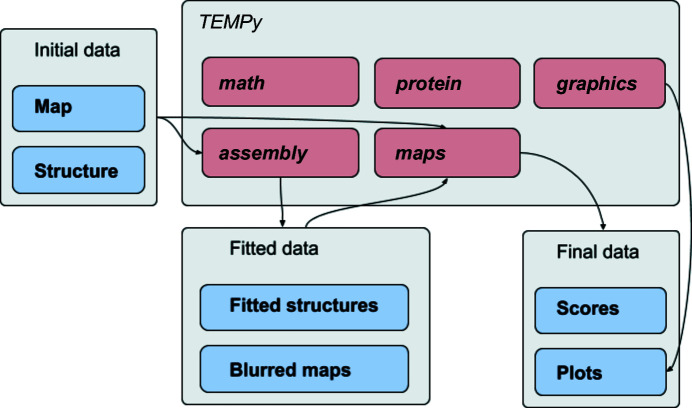
A generic pipeline that makes use of *TEMPy*2 routines from most modules. Not shown are the initial loading of the map and structure object from the *protein* and *maps* modules, as well as the internal use of the *math* module.

The different routines within *TEMPy*2 are mostly independent and can be combined in any user-defined way, although they tend to generate and manipulate objects that are most easily created by the input/output routines from within *TEMPy*2 (Fig. 3 ▸). To further motivate and clarify the potential uses for these routines, we now provide detailed, concrete examples.

### 3.1. Map-to-map alignment   

Optimizing the alignment between two maps is an important task, although often hidden within a larger pipeline. The optimization is usually carried out with respect to a given scoring function, such as those described previously.

*TEMPy*2 contains both local and global optimization routines. A local optimization routine iteratively improves upon a given initial state until no further improvement can be made. This is usually relatively quick (for example, the optimization of the position and orientation of a 36 × 27 × 22 voxel map with respect to a reference map takes 16.3 s for 100 steps on a single core of a 2.9 GHz Intel i9 processor; this can be invoked with the local_align.py script and two maps). Two new local optimization routines have been implemented in *TEMPy*2: a Monte Carlo search, with a small step size, and an expectation–maximization search. Global optimization will usually involve testing a (large) number of starting points, potentially running a local optimization and then returning the best found solution. For global optimization two options are available: the previously implemented genetic algorithm, *γ-TEMPy* (Pandurangan *et al.*, 2015 ▸), which is presented further in detail below, and a new quasi-Monte Carlo scheme that generates samples across the entire search space without producing repeated points. While similar to a grid search, it does not require a grid level to be provided.

**Figure 4:**
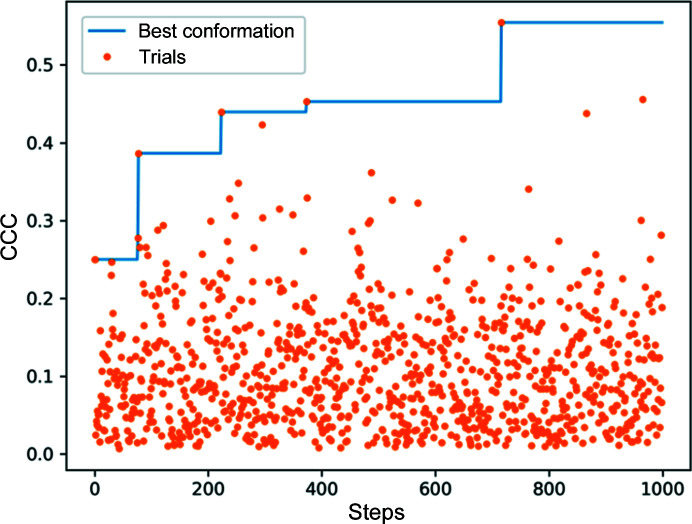
Change in CCC during a global optimization run. The blue line shows the highest CCC conformation found, with the orange dots showing the CCC of the trial conformation sampled during the run.

The global optimization process can be slower than a local optimization, although it is less reliant on a good initial starting point. Both types of searches and combinations of them are possible with *TEMPy*2. By default, we run a fast initial global search and then perform a local search afterwards. Fig. 4 ▸ provides an example of the change in CCC during a global search.

The local Monte Carlo optimization routine is based on a standard Metropolis criterion (Metropolis *et al.*, 1953 ▸), by default using CCC. Starting from an initial position, the CCC with respect to the reference map is computed and optimized according to this criterion (Cragnolini *et al.*, in preparation).

The expectation–maximization scheme proceeds by iteratively computing the most likely position of the map centre, assuming the map to be optimized as an estimate of the reference map (Kawabata, 2008 ▸).

### 3.2. Difference map   

When two proteins have been resolved, for example with or without a ligand present, it is useful to characterize the difference in density resulting from the change. To do this, a method is needed to compute this difference between maps. This is usually performed after map-to-map fitting as described above. The maps are first scaled based on their resolution-dependent amplitude falloffs and the difference is then calculated (Joseph *et al.*, 2020 ▸).

**Figure 5:**
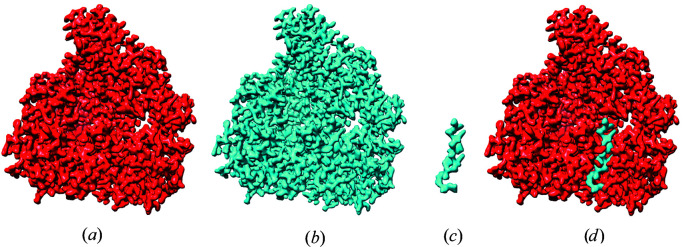
Example of difference mapping with *TEMPy*2 generated from PDB entry 6kle with and without the presence of a ligand. (*a*) Map for unbound protein. (*b*) Map for ligand-bound protein. (*c*) Difference map. (*d*) Initial maps superimposed to show the ligand placement.

Fig. 5 ▸ shows a difference-mapping example generated from PDB entry 6kle with and without the presence of a ligand. The difference between the two maps helps to identify the position and shape of a ligand and its interaction pattern (Locke *et al.*, 2017 ▸; Peña *et al.*, 2020 ▸). The difference-map protocol can identify larger differences between structures, such as conformational changes.

### 3.3. Map–structure fit optimization   

To better understand the biochemical nature of a system of interest, a known or pre-calculated atomistic model is often fitted within a cryo-EM density map (unless the atomic positions can be determined directly from the density). The fitting task is important to ensure that the model properly matches the features of the map. *TEMPy*2 incorporates several routines that can be used together to compute and optimize the fit between such a map and model.

Firstly, a map is obtained from the model by computing the sum of intensities of Gaussian functions centred on each atom in the model, with an appropriate spread (a combined effect of the *B* factor, sigma factor and resolution) and maximum intensity (corresponding to the electronic number of the atom). The optimization then proceeds with the same protocol as outlined for a map-to-map optimization.

### 3.4. *γ-TEMPy*   

*γ-TEMPy* is an optimization method designed to produce assemblies of multi-component protein systems that best fit to a given map. A genetic algorithm is used to refine the search and eventually produce well fitted models (Pandurangan *et al.*, 2015 ▸). The gamma-tempy.py script can be invoked to run a similar fitting procedure starting from a given map and structure.

**Figure 6:**
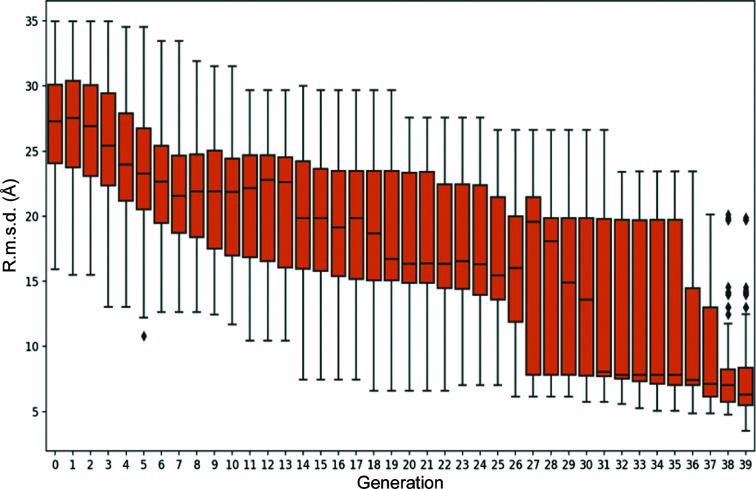
Evolution of the C^α^ r.m.s.d. of the trial conformations in the population. The C^α^ r.m.s.d. is computed against the correct conformation, which is not available during the optimization run. The fit quality during optimization is evaluated using the CCC.

An example of the change in the C^α^ r.m.s.d. of the fitted components during optimization with respect to the corresponding crystal structure (PDB entry 1cs4) is shown in Fig. 6 ▸. An iterative improvement of the fit of the generated models with respect to the map is apparent, with worse models being eliminated and better models being kept and improved in each generation.

### 3.5. Model assessment   

**Figure 7:**
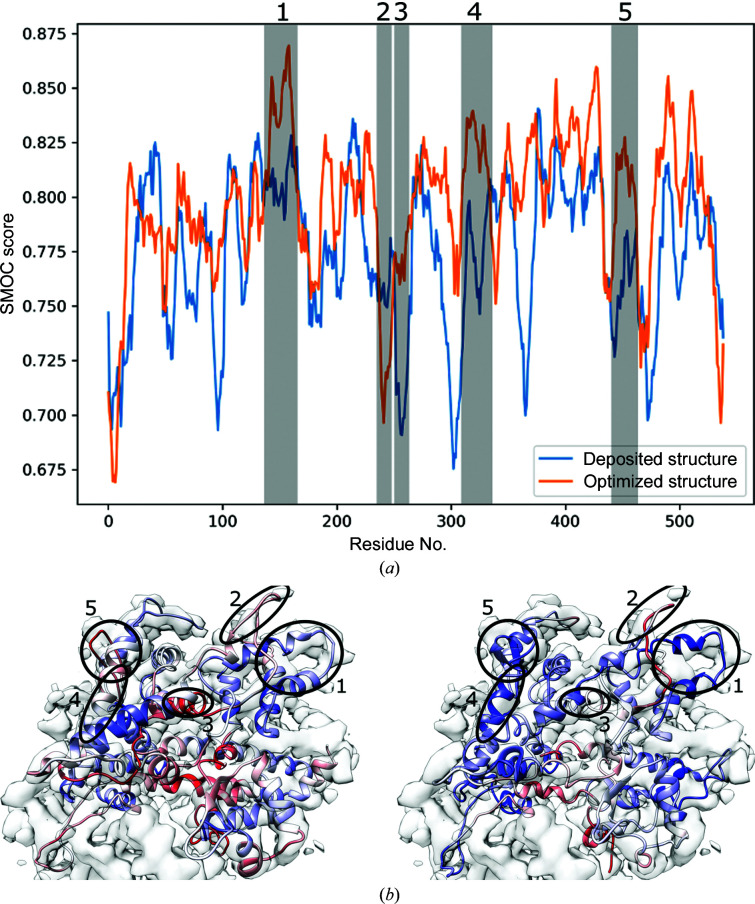
(*a*) SMOC_f_ profile computed for chain *C* of RNA polymerase III (PDB entry 5fj8) against the experimentally determined map (EMDB entry EMD-3178) (Hoffmann *et al.*, 2015 ▸) before (blue) and after (orange) optimization. Regions of significant changes are shaded and numbered. (*b*, *c*) Deposited (left) and optimized (right) structures of chain *O*, aligned with the map, coloured by SMOC score. Blue represents higher scores; red represents lower scores. The circled regions correspond to those in (*a*).

Assuming that we have a model fitted within a map (for example using the routines presented above or given as an input from another source), we may be interested in quantifying not only the global fit (Fig. 6 ▸) but also its local quality. Fig. 7 ▸ illustrates the quality of fit of two conformations (the deposited structure and an optimized structure refined with *Flex-EM*; Topf *et al.*, 2008 ▸) computed with SMOC_f_ (Joseph *et al.*, 2017 ▸) on chain *O* of RNA polymerase III (PDB entry 5fj8) against the experimentally determined map (EMDB entry EMD-3178) (Hoffmann *et al.*, 2015 ▸). The sequence-based score shows how the fit quality has changed across different regions of the chain before (blue) and after (orange) optimization. A similar profile could be obtained with SCCC rather than SMOC. The score_smoc.py script can be invoked to run a similar analysis on a structure and map, or the SMOC method of the scoring module can be used to the same effect programmatically in Python.

### 3.6. Integration   

*CCP-EM* (Burnley *et al.*, 2017 ▸) provides a software suite integrating many popular cryo-EM tools in a common interface. The following *TEMPy*2 routines are available through the suite: CCC, MI, SCCC, SMOC_d_, SMOC_f_ and difference maps.

## 4. Conclusion   

In this manuscript, we have presented recent advances in the *TEMPy*2 package and workflows illustrating its use. *TEMPy*2 allows a user to easily load maps and structures, and perform a variety of map and model processing, optimization and validation tasks that can be entirely customized. The Python package and class structure can be further extended to develop code on top of the core routines of *TEMPy2*, or higher level functions can be used for more routine tasks. The new version of the software provides stronger testing to ensure consistency of results, as well as methodological developments to improve and assess the quality of the fit of structure to maps. Documentation and code are available to download at http://tempy.ismb.lon.ac.uk and include a set of examples.

## Figures and Tables

**Table 1:** The following table summarizes the different global scores available in *TEMPy*2

Score	Shorthand	Reference
Cross-correlation coefficient	CCC	Roseman (2000 ▸)
Mutual information	MI	Vasishtan & Topf (2011 ▸)
Least-square fit	LSF	Vasishtan & Topf (2011 ▸)
Normal vector score	NV	Vasishtan & Topf (2011 ▸)
Chamfer distance	CD	Vasishtan & Topf (2011 ▸)
Envelope score	ENV	Vasishtan & Topf (2011 ▸)
